# Evolutions in rectal cancer MRI staging and risk stratification in The Netherlands

**DOI:** 10.1007/s00261-021-03281-8

**Published:** 2021-10-04

**Authors:** Nino Bogveradze, Najim el Khababi, Niels W. Schurink, Joost J. M. van Griethuysen, Shira de Bie, Gerlof Bosma, Vincent C. Cappendijk, Remy W. F. Geenen, Peter Neijenhuis, Gerald Peterson, Cornelis J. Veeken, Roy F. A. Vliegen, Monique Maas, Max J. Lahaye, Geerard L. Beets, Regina G. H. Beets-Tan, Doenja M. J. Lambregts

**Affiliations:** 1 Department of Radiology, The Netherlands Cancer Institute, P.O. Box 90203, 1006 BE Amsterdam, The Netherlands; 2 GROW School for Oncology & Developmental Biology, University of Maastricht, Maastricht, The Netherlands; 3 Department of Radiology, Acad. F. Todua Medical Center, Research Institute of Clinical Medicine, Tbilisi, Georgia; 4 Department of Radiology, Deventer Ziekenhuis, Deventer, The Netherlands; 5 Department of Radiology, Elisabeth Tweesteden Hospital, Tilburg, The Netherlands; 6 Department of Radiology, Jeroen Bosch Hospital, ‘s Hertogenbosch, The Netherlands; 7 Department of Radiology, Northwest Clinics, Alkmaar, The Netherlands; 8 Department of Surgery, Alrijne Hospital, Leiderdorp, The Netherlands; 9 Department of Radiology, Spaarne Gasthuis, Haarlem, The Netherlands; 10 Department of Radiology, IJsselland Hospital, Capelle aan den IJssel, The Netherlands; 11 Department of Radiology, Zuyderland Medical Center, Heerlen, The Netherlands; 12 Department of Surgery, The Netherlands Cancer Institute, Amsterdam, The Netherlands; 13 Institute of Regional Health Research, University of Southern Denmark, Odense, Denmark

**Keywords:** Rectal neoplasms, Neoplasm staging, Risk assessment, Magnetic resonance imaging

## Abstract

**Purpose:**

To analyze how the MRI reporting of rectal cancer has evolved (following guideline updates) in The Netherlands.

**Methods:**

Retrospective analysis of 712 patients (2011–2018) from 8 teaching hospitals in The Netherlands with available original radiological staging reports that were re-evaluated by a dedicated MR expert using updated guideline criteria. Original reports were classified as “free-text,” “semi-structured,” or “template” and completeness of reporting was documented. Patients were categorized as low versus high risk, first based on the original reports (high risk = cT3-4, cN+, and/or cMRF+) and then based on the expert re-evaluations (high risk = cT3cd-4, cN+, MRF+, and/or EMVI+). Evolutions over time were studied by splitting the inclusion period in 3 equal time periods.

**Results:**

A significant increase in template reporting was observed (from 1.6 to 17.6–29.6%; *p* < 0.001), along with a significant increase in the reporting of cT-substage, number of N+ and extramesorectal nodes, MRF invasion and tumor-MRF distance, EMVI, anal sphincter involvement, and tumor morphology and circumference. Expert re-evaluation changed the risk classification from high to low risk in 18.0% of cases and from low to high risk in 1.7% (total 19.7%). In the majority (17.9%) of these cases, the changed risk classification was likely (at least in part) related to use of updated guideline criteria, which mainly led to a reduction in high-risk cT-stage and nodal downstaging.

**Conclusion:**

Updated concepts of risk stratification have increasingly been adopted, accompanied by an increase in template reporting and improved completeness of reporting. Use of updated guideline criteria resulted in considerable downstaging (of mainly high-risk cT-stage and nodal stage).

**Graphic abstract:**

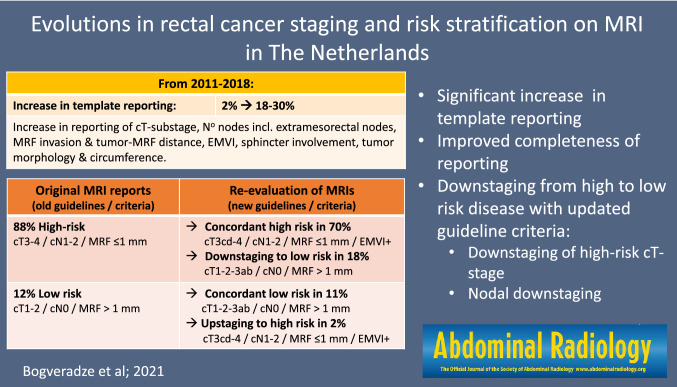

## Introduction

MRI is routinely used to stratify rectal cancer patients for differentiated treatments based on the presence (or absence) of known high-risk features. Traditionally, the main high-risk features used in clinical guidelines to stratify patients for neoadjuvant treatment included cT3-4 disease, tumor invasion of the mesorectal fascia (MRF), and node-positive (cN +) disease [1–5].

In this setup, borderline cT2-3 tumors posed a diagnostic challenge as—despite technological improvements in high-resolution imaging—it remains difficult to distinguish T2 tumors with desmoplasia from tumor stranding in early T3 tumors [6]. Recently, the clinical significance of this distinction has been questioned as several pathology studies have demonstrated that it is mainly T3 tumors with more extensive invasion (> 5 mm) beyond the rectal wall that constitute the group with a high risk of locoregional recurrence [7–11]. The Mercury study group showed that high-resolution MRI can accurately determine the depth of extramural invasion [12] and a report by Taylor et al. showed that, by doing so, MRI can accurately identify tumors with a low-risk cT-stage (cT1-2 and cT3 with < 5 mm perirectal invasion) that can safely be managed by surgery only [13]. This subdivision of cT-stage according to the depth of invasion has meanwhile been adopted for risk stratification in several guidelines [1, 3, 14].

The staging of lymph nodes has also evolved during the last decade. Although the clinical significance of node-positive disease (as assessed on imaging) is questioned by some [13, 15], it is still included as a treatment determinant in many guidelines [1–5]. Traditionally, positive nodes were mainly determined using size criteria, resulting in insufficient sensitivities and specificities ranging between only 55 and 78% [16, 17]. More recently, adverse morphologic features (heterogeneous signal, round shape, irregular border contour) have been adopted into guidelines as additional criteria to diagnose cN+ nodes which have improved the performance of MRI for nodal staging [3, 14, 18].

A third development has been the increased acknowledgement of extramural vascular invasion (EMVI) as a relevant prognostic risk factor. Although not (yet) adopted in most guidelines as a main treatment determinant, there have been several reports showing that the presence of MRI-detected EMVI is a poor prognostic factor associated with an increased risk for metastases and impaired disease-free survival [15, 19, 20]. In the most recent consensus guidelines on rectal MRI published by the European Society of Gastrointestinal and Abdominal Radiology (ESGAR) it is now recommended to routinely include EMVI in the radiological staging report as a factor entailing more high-risk disease stage [14].

Such developments warrant more precise radiological reporting and increase the need for structured reporting where all key elements to allow informed clinical decision making are sufficiently described. As with any new developments and guidelines updates, it takes time before these are fully acknowledged and implemented into general clinical practice. The aim of this study was to retrospectively analyze how the MRI reporting of rectal cancer has evolved over a period of ± seven years in The Netherlands (following guideline updates) by assessing trends in the use of structured reporting, evaluating how novel risk concepts such as cT3 substaging, updated nodal staging criteria, and EMVI have been adopted into routine reporting, and exploring its potential impact on treatment stratification.

## Methods

### Patient selection

This study was performed as a side project of an ongoing IRB-approved retrospective multicenter imaging study focused on MRI for risk and response assessment in rectal cancer. Due to the retrospective nature of the study, informed consent was waived. As part of this multicenter project the primary staging of MRIs including radiological staging reports, treatment specifics (type of surgery and type of neoadjuvant treatment, if any), and clinical outcome data of 1426 patients with biopsy-proven rectal adenocarcinoma were previously collected, originating from 10 Dutch medical centers (1 university hospital, 8 large teaching hospitals, and 1 comprehensive cancer center). As part of this previous study project, the MRI examinations of a subset of the collected study patients were re-evaluated by a single dedicated MRI expert (DMJL with > 10 years of experience in reading rectal MRI) from the principal investigating (PI) center according to the staging template published in the most recent ESGAR consensus guidelines on rectal MRI from 2018 [14]. The reader was blinded for the original staging reports and any other clinical information regarding treatment or treatment outcome. For the current study, we collected from this dataset all patients originating from the eight teaching hospitals in the cohort who fulfilled the following inclusion criteria: (a) availability of the original primary staging report and (b) availability of a second re-evaluation report by the MRI expert from the PI center. As the aim of this study was to evaluate staging trends and effects in a general hospital setting, only patient cases from the eight general teaching hospitals were included and cases from the academic and comprehensive cancer center (both expert referral centers for rectal cancer) were excluded.

### Classification of type and completeness of reporting

A second independent observer other than the MR expert who performed the imaging re-evaluations (NB) reviewed the original radiological staging reports and classified the type of reporting as “free-text,” “semi-structured,” or “template.” Reports were categorized as free-text when including only prose descriptions without any specific subheadings (apart from “findings” and/or “conclusion”) or standardized reporting items. Reports were classified as semi-structured when the report was organized using subheadings, including for example “tumor” (or “tumor stage”) and “nodes”(or “nodal stage”). Reports were classified as template reports if the report included an itemized list of reporting items, e.g., morphology, location, T-stage, N-stage, MRF, sphincter involvement, EMVI, etc. In addition, completeness of reporting was documented for each staging report by assessing for each item listed in the ESGAR structured report template whether it was explicitly reported, not explicitly reported but otherwise derivable from the report, or not reported at all.

### Risk classification

All patients in the cohort were classified as low versus high risk, based on the original staging reports and based on the re-evaluations performed at the PI center, respectively, using the criteria detailed in Table 1. For the original staging reports, patients were classified as high risk in the case of the presence of either of the following: ≥ cT3 stage, cN1-2 stage, tumor-MRF distance of ≤ 1 mm; in line with clinical guidelines that are applied during the main part of the study inclusion period [1–5, 21]. For the re-evaluation reports performed at the PI center, patients were classified as high risk in case of ≥ cT3cd stage, cN1-2 stage, tumor-MRF distance of ≤ 1 mm, and/or presence of extramural vascular invasion (EMVI); in line with current guideline updates [3, 14].

**Table 1:** Criteria used for risk classification

	Original reports (“old” guidelines)	Re-evaluation (“updated” guidelines)
Low risk	cT1-2	cT1-2-3ab
	cN0	cN0
	Tumor-MRF distance > 1 mm	Tumor-MRF distance > 1 mm
High risk	cT3-4	cT3cd-4
	cN1-2	cN1-2
	Tumor-MRF distance ≤ 1 mm	Tumor-MRF distance ≤ 1 mm
	-	EMVI +

cT3a =  < 1 mm invasion beyond rectal wall

cT3b = 1–5 mm invasion beyond rectal wall

cT3c = 5–15 mm invasion beyond rectal wall

cT3d =  > 15 mm invasion beyond rectal wall

**Table 2:** Type and completeness or reporting

		Total *N* = 712	Part 1 Q4 2011–Q1 2014 *N* = 191	Part 2 Q1 2014–Q1 2016 *N* = 334	Part 3 Q2 2016–Q2 2018 *N* = 187	*p * value
*Type of reporting*						
Report Type	Free text	77.8% (554)	97.9% (187)	67.1% (224)	76.5% (143)	<0.001
	Semi-structured	3.2% (23)	0.5% (1)	3.3% (11)	5.9% (11)	
	Structured (template)	19.0% (135)	1.6% (3)	29.6% (99)	17.6% (33)	
*Items included in report*						
1 Morphology	Lesion type reported (polyp, semi-annular, annular)	66.6% (474)	51.8% (99)	67.4% (225)	80.2% (150)	<0.001
	Not reported	33.4% (238)	48.2% (92)	32.6% (109)	19.8% (37)	
	Tumor type reported (solid, mucinous)	6.6% (47)	2.1% (4)	8.4% (28)	8.0% (15)	0.013
	Not reported	93.4% (665)	97.9% (187)	91.6% (306)	92.0% (172)	
2 Tumor circumference	Specified as from … to … o’clock	12.4% (88)	5.8% (11)	15.6% (52)	13.4% (25)	<0.001
	Only prose description (ventral/dorsal/lateral)	12.2% (87)	19.9% (38)	10.8% (36)	7.0% (13)	
	Not reported	75.4% (537)	74.3% (142)	73.7% (246)	79.7% (149)	
3 Height	Reported as measurement from ARJ/anal verge	92.6% (659)	88.5% (169)	92.8% (310)	96.3% (180)	0.018
	Only prose description (low/mid/upper)	5.6% (40)	7.3% (14)	6.0% (20)	3.2% (6)	
	Not reported	1.8% (13)	4.2% (8)	1.2% (4)	0.5% (1)	
4 Length	Reported in cm/mm	90.3% (643)	87.4% (167)	91.0% (304)	92.0% (172)	0.274
	Not reported	9.7% (69)	12.6% (24)	9.0% (30)	8.0% (15)	
5 cT-stage	Reported incl. substaging (incl. cT3abcd, cT4ab)	22.8% (162)	2.1% (4)	19.8% (66)	49.2% (92)	<0.001
	Reported without substaging (cT1234)	65.6% (467)	70.2% (134)	73.4% (245)	47.1% (88)	
	Not explicitly mentioned but can be derived from prose description*	10.3% (73)	25.1% (48)	5.7% (19)	3.2% (6)	
	Not reported	1.4% (10)	2.6% (5)	1.2% (4)	0.5% (1)	
6 Anal sphincter involvement	Reported	7.9% (56)	3.7% (7)	8.4% (28)	11.2% (21)	0.004
	Not reported	92.1% (656)	96.3% (184)	91.6% (306)	88.7% (166)	
	in low tumors	33.7% (240)	42.9% (82)	31.4% (105)	28.3% (53)	
	in mid/high tumors (N/A)	58.4% (416)	53.4% (102)	60.2% (201)	60.4% (113)	
7 MRF invasion	Reported	81.4% (580)	73.3% (140)	82.9% (277)	87.2% (163)	0.032
	Not reported	16.6% (118)	24.1% (46)	15.0% (50)	11.7% (22)	
	in cT3-4 tumors^#^	8.6% (61)	11.0% (21)	7.5% (25)	8.0% (15)	
	in cT1-2 tumors (N/A)^#^	7.4% (53)	12.6% (24)	6.9% (23)	3.2% (6)	
	Inconclusive**	2.0% (14)	2.6% (5)	2.1% (7)	1.1% (2)	
8 Tumor-MRF margin	Reported	59.7% (426)	48.7% (93)	65.6% (219)	61.0% (114)	0.004
	Not reported	40.2% (286)	51.3% (98)	34.5% (115)	39.0% (73)	
	in cT3-4 tumors^#^	28.2% (201)	36.1% (69)	22.2% (74)	31.0% (58)	
	in cT1-2 tumors (N/A)^#^	11.4% (81)	14.7% (28)	11.7% (39)	7.5% (14)	
9 cN-stage	Reported incl. substaging (cN0/cN1abc/cN2ab)	1.3% (9)	0.5% (1)	2.4% (8)	0% (0)	<0.001
	Reported as cN0/N1/N2	74.2% (528)	58.1% (111)	74.6% (249)	89.8% (168)	
	Reported as cN-/N+	6.3% (45)	1.6% (3)	10.8% (36)	3.2% (6)	
	Not explicitly mentioned but can be derived from prose description of number of suspicious nodes	11.1% (79)	19.4% (37)	9.0% (30)	6.4% (12)	
	Not reported	7.2% (51)	20.4% (39)	3.3% (11)	0.5% (1)	
10 Number of N+ nodes (in cN+ cases)	Reported	54.9% (391)	41.9% (80)	57.5% (192)	63.6% (119)	0.014
	Not reported	12.9% (92)	15.7% (30)	13.5% (45)	9.1% (17)	
11 Total number of nodes	Reported	9.3% (66)	10.5% (20)	8.1% (27)	10.2% (19)	0.588
	Not reported	90.7% (646)	89.5% (171)	91.9% (307)	89.8% (168)	
12 Extramesorectal (lateral) nodes	Reported	52.2% (372)	27.7% (53)	63.5% (212)	57.2% (107)	<0.001
	Not reported	47.8% (340)	72.3% (138)	36.6% (122)	42.8% (80)	
	in cN+ cases^#^	27.7% (197)	39.3% (75)	21.0% (70)	27.8% (52)	
	in cN- cases (N/A)^#^	14.5% (103)	15.7% (30)	13.5% (45)	15.0% (28)	
13 Tumor deposits	Reported	1.3% (9)	0.5% (1)	1.5% (5)	1.6% (3)	0.561
	Not reported	98.7% (703)	99.5% (190)	98.5% (329)	98.4% (184)	
14 EMVI	Reported	28.0% (200)	4.7% (9)	36.2% (121)	37.4% (70)	<0.001
	Not reported	72.0% (512)	95.3% (182)	63.8% (213)	62.5% (117)	
	in cT3-4 tumors^#^	54.4% (387)	72.8% (139)	46.4% (155)	49.7% (93)	
	in T1-2 tumors (N/A)^#^	16.3% (116)	19.9% (38)	16.5% (55)	12.3% (23)	

*Examples of prose descriptions from which cT-stage could be derived: “Tumor limited to bowel wall,” “Tumor extending into perirectal fat,” “Tumor growing into peritoneum,” etc

**MRF invasion was categorized as inconclusive in case of unclear descriptions such as “close margin”

#In some cases, sub-categorization was not feasible not feasible due to missing information on cT-stage or cN-stage, respectively.

### Statistical analyses

Statistical analyses were performed using IBM SPSS Statistics version 25.0 (IBM Corporation, Armonk, NY, USA). Data were primarily analyzed using descriptive statistics where categorical or dichotomous variables were recorded as absolute numbers with percentages. Trends in completeness of reporting over time were analyzed by dividing the cohort into 3 equal time periods of ±26 months (12/2011 to 2/2014; 3/2014 to 3/2016; 4/2016 to 6/2018). For intergroup comparison of categorical and dichotomous outcomes, the Pearson Chi-Square test was used. *p* values < 0.05 were considered statistically significant.

## Results

### Patient cohort

From the initial cohort of 1426, *n* = 712 patient cases could be included (63.6% male, median age 66, range 26–94 years), for whom both the original primary staging reports and re-evaluation reports (using updated guideline criteria) were available. These patients included 95 (13.3%) patients who were treated with direct surgery, 61 (8.6%) patients who underwent short-course radiotherapy (5 × 5 Gy) followed by surgery, and 556 (78.1%) patients who underwent a long course of neoadjuvant treatment (i.e., chemoradiotherapy or 5 × 5 Gy with an extended waiting interval to surgery).

### Type and completeness or reporting

Table 2 demonstrates evolutions in the type and completeness of reporting over time. During the study inclusion period, a significant decrease in free-text reporting and corresponding increase in template reporting was observed, with template reports constituting 17.6%-29.6% of all reports in the second and third part of the study period (vs. only 1.6% in the first period; *p* < 0.001). Items that were consistently reported in ≥ 80% of reports (regardless of the study period) included tumor height, length, cT- stage, and cN-stage (as cN0/cN +). A significant increase over time was observed for the reporting of cT-substage (cT3abcd and cT4ab), number of suspicious lymph nodes (incl. substaging of N-stage as cN0/1/2 and the presence of suspicious extramesorectal lymph nodes), MRF invasion, distance between tumor and MRF, EMVI, anal sphincter invasion, tumor morphology, and tumor circumference.

### Risk stratification

**Fig. 1:**
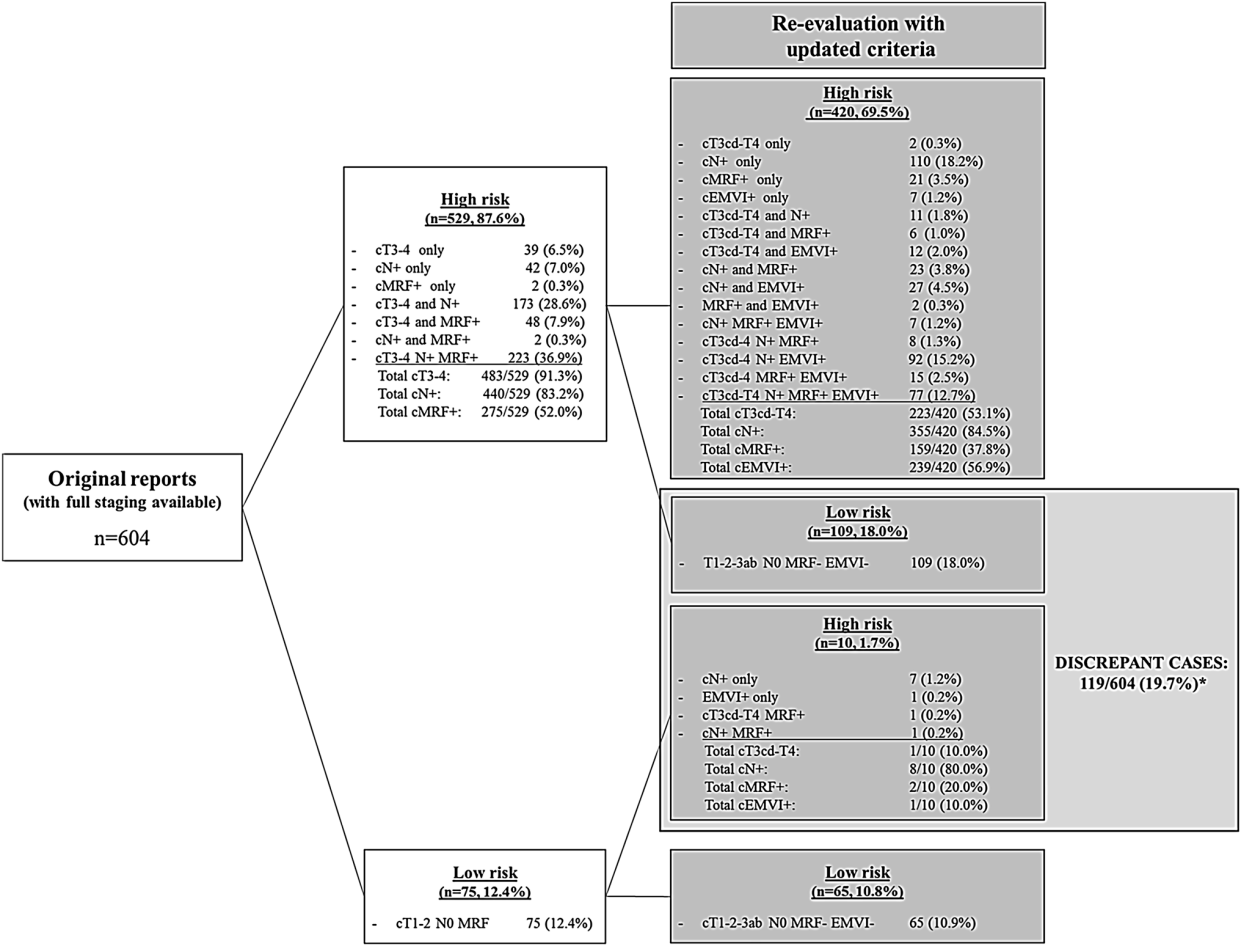
Effect after re-evaluation of study cases using updated staging criteria on classification of patients into low risk versus intermediate/high risk. * Note, in 11 out of the 119 discrepant cases, the change in risk classification was clearly due to interpretation differences (rather than use of updated criteria) between the original staging reports and the expert-re-evaluation: 7 cases originally staged as cT4 were downstaged to low-risk cT12-3ab disease, 2 cases originally staged as cT1-2 MRF + were re-evaluated as cT1-2 MRF-, and 2 cases originally staged as cT1-2 MRF- were re-evaluated as cT3 MRF + . This left a total of 108/604 = 17.9% remaining discrepant cases

**Fig. 2:**
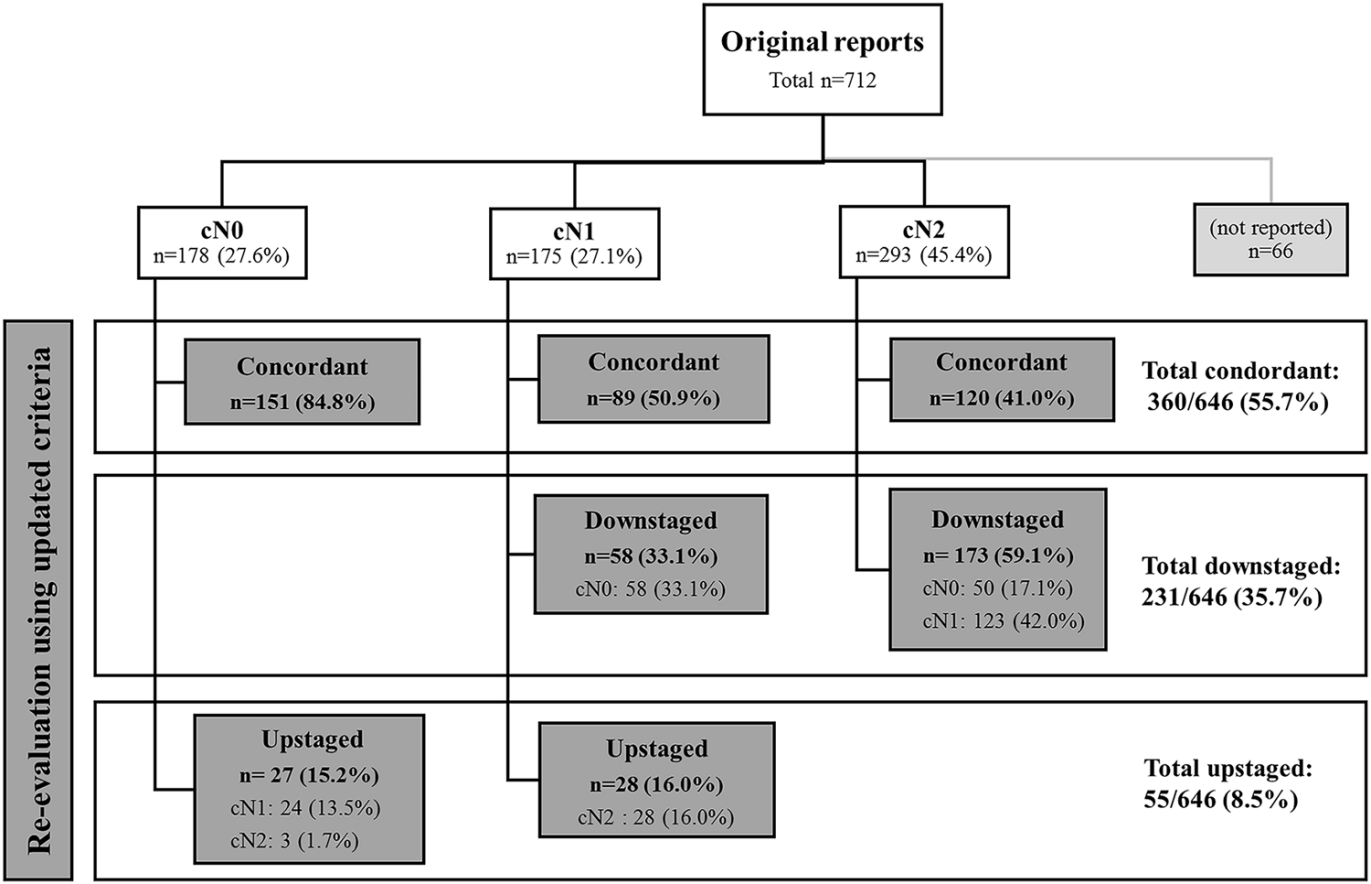
Changes in nodal stage after re-evaluation of cases using updated nodal staging criteria

Figure 1 demonstrates the categorization of patients into low risk versus high risk according to the original staging reports and shows how this categorization was affected after re-evaluation using updated staging criteria (including cT-substaging as cT3ab versus cT3cd, updated nodal staging criteria, and implementation of EMVI). These results could be analyzed for 604 out of the 712 study patients; for the remaining 108 patients one or more required staging items (cT-stage, cN-stage or MRF invasion) were missing from the original staging reports. Re-evaluation of the patient cases changed the risk classification from high to low risk in 109/604 (18.0%) cases and from low to high risk in 10/604 (1.7%) cases (total 119; 19.7%). In 11 out of these 119 cases, the change in risk classification was mainly due to interpretation differences between the original staging reports and the expert-re-evaluation, including downstaging of cT4 tumors to low-risk cT12-3ab disease and conversion from MRF + to MRF− stage or vice versa. The remaining 108 cases (17.9%) with a change in risk classification were mainly attributable to changes in the classification of high-risk cT-stage and changes in cN-stage. Figure 2 provides a more detailed overview of the changes in cN-stage (using updated nodal staging criteria), which resulted in nodal downstaging in 35.7% of cases and nodal upstaging in 8.5% of cases. In the remaining 55.7% of cases, cN-stage remained concordant.

## Discussion

This study demonstrates that novel concepts of risk stratification such as cT3 substaging and reporting of EMVI have increasingly been adopted in radiological reports in MRI reporting in The Netherlands from 2011 to 2018. During the same period, we have observed a clear increase in the use of structured reporting templates and an overall trend towards improved completeness of reporting. When retrospectively applying updated criteria for risk stratification, as adopted by recent guidelines, this might have resulted in a change in risk status in approximately 18% of patients in our cohort.

The main factors that changed the risk stratification were a reduction in the number of patients classified as high risk based on cT-stage and a reduced number of patient staged as node-positive. Of the 483 patients staged as cT3-4 in the original reports, only 223 (46.2%) were categorized as having a high-risk cT-stage (≥ cT3cd) when applying updated criteria for cT-staging where only tumors with an invasion depth of > 5 mm beyond the rectal wall are considered high-risk tumors [1, 3, 14]. In the remaining 53.8% of cases, re-evaluation including cT-substaging revealed a low-risk cT-stage (≤ cT3ab), which—provided that no other risk criteria are present—may be treated surgically without the necessity for neoadjuvant treatment [13], though in some countries and guidelines (particularly in the United States) it remains routine practice to give neoadjuvant treatment to any cT3 tumor, regardless of invasion depth.

The high proportion (35.7%) of nodal downstaging can probably be attributed to the fact that images were all assessed by a dedicated reader with consistent use of the nodal staging criteria as detailed in the structured report template proposed by ESGAR, while the original reports were generated by a variety of radiologists from the participating centers and likely with varying criteria. Although we obviously cannot be sure which criteria were used by these radiologists, it is likely to assume that at least part of the scans were assessed using traditional (size-based) criteria considering that a considerable proportion of the cohort originated from < 2014, i.e., before updated criteria on nodal staging including nodal morphology were adopted by the 2014 updates of the Dutch guidelines and before the most recent ESGAR consensus guidelines were published. As demonstrated in the previous literature, use of size-based criteria may result in substantial nodal overstaging [16, 17]. A population-based study of 14.018 patients in The Netherlands treated for rectal cancer between 2009 and 2014 showed a substantial decrease in the use of preoperative radiotherapy (versus surgery only) after implementation of the Dutch national guideline updates in 2014, which was accompanied by a marked increase in the specificity of MRI for nodal staging (from 62.9% in 2013 to 73.2% in 2014), indicating a decrease in nodal overstaging [22]. A more recent Dutch study by Detering et al. covering the period 2011–2017 (total 21.385 patients) confirmed a significant decrease in the use of preoperative radiotherapy for early-stage tumors in the period following the 2014 guideline updates. Again, the authors suggested that this decrease may at least in part be contributed to the updated guidelines on nodal staging that increased the threshold to diagnose nodes as malignant on MRI [23]. According to the ESGAR (and Dutch) guidelines, only nodes with a short-axis diameter of ≥ 9 mm are immediately staged as N + based on size only. For nodes with a short-axis of 5–8 mm or < 5 mm, two or even three additional morphologically suspicious criteria (round shape, irregular border, heterogeneous signal) are required in order to call a node malignant [3, 14]. Our results confirm trends shown in the previous population studies that this approach leads to substantial downstaging of nodes, compared to use of traditional (size-based) criteria.

With respect to EMVI, we observed that this is a risk factor that is increasingly being reported in routine practice, reflecting an increased awareness of EMVI as a relevant prognostic feature to include in routine reporting. While in the first part of the study period (2011–2014) EMVI was only reported in < 5% of the cases, this number increased significantly to 37.4% in the final years of the study period up to 2018. The cases where EMVI was not reported included a substantial number of cT1-2 cases where reporting of EMVI will in most cases be considered as less or irrelevant. Although EMVI is increasingly acknowledged and adopted in structured reporting templates as a relevant prognostic risk factor, it has not (yet) been widely implemented as a main treatment determinant in current clinical guidelines. Looking at our current results, EMVI by itself would have had only a minor additional impact on treatment decision making, as the presence of EMVI almost exclusively went hand in hand with the presence of other high-risk features (cT3cd-4 stage, cN + stage, cMRF + stage). Only in 7 cases (1.2%) EMVI was the only high-risk feature present on MRI.

Finally, our study showed a vast increase in the use of structured (template) reporting, as well as improved completeness of reporting for several items including MRF invasion, anal sphincter invasion, lateral nodal involvement, and tumor morphology. These findings are likely related to one another and in line with the previous reports demonstrating that template reports are superior to free-text reports in terms of completeness of reporting [24, 25]. Additional benefits of structured reporting described in the literature include improved clarity and consistent use of terminology across practices which in turn guarantees better communication in imaging [26–28]. Overall, it has been suggested that implementation of structured reporting templates can improve the quality of MRI reporting for rectal cancer compared to free-text formats, and leads to higher satisfaction levels from referring clinicians [29, 30]. Somewhat surprisingly, the percentage of structured reports decreased in the third part of the study period, after an initial steep increase in the second part of the study. This can be attributed to the fact that two of the centers in the cohort with the highest rate of structured reporting were relatively underrepresented in the third part of the study period.

Our study has some limitations, in addition to its retrospective study design. As before mentioned, all re-evaluations using updated staging criteria were done by single experienced rectal MRI reader, whereas original interpretations and reports were done by multiple readers as part of routine clinical practice. We have no detailed information on the experience level of these readers and it is conceivable that at least part of the discrepant findings after re-evaluation of the images can be attributed to variations in reader experience rather than variations in guidelines and criteria used. Along this line, we have no way of knowing which criteria were used by the various radiologists while performing their original staging reports. However, we do know that updated guideline criteria (in particular for nodal staging) were not yet available or published during the early years of the study period, and therefore likely not used.

In conclusion, this study shows that updated concepts of risk stratification in rectal cancer such as cT3 substaging, revised criteria for nodal staging, and reporting of EMVI have increasingly been adopted during the last decade in teaching hospitals in The Netherlands. This was accompanied by increased use of template reporting and overall improved completeness of reporting. Use of updated guideline criteria resulted in significant downstaging of high-risk cT-stage and nodal stage compared to the original reports. This might, in retrospect, have changed risk (and consequently treatment) stratification in approximately 18% of patients in our cohort. Our results support the use of template reporting using consistent (guideline-based) imaging criteria to further improve consistency, clarity, and completeness of reporting in the future.
